# Meat quality and lipid fatty acid profile from wild thrush (*Turdus philomelos*), woodcock (*Scolopax rusticola*) and starling (*Sturnus vulgaris*): a preliminary comparative study

**DOI:** 10.1186/s12944-020-01300-z

**Published:** 2020-06-02

**Authors:** Simona Tarricone, Maria Antonietta Colonna, Carlo Cosentino, Francesco Giannico, Marco Ragni

**Affiliations:** 1 Department of Agro-Environmental and Territorial Sciences, University of Bari ‘Aldo Moro’, 70125 Bari, Italy; 2 School of Agricultural, Forest, Food, and Environmental Sciences, University of Basilicata, 85100 Potenza, Italy

**Keywords:** Gamebirds, Meat, Fatty acids, Thrush, Woodcock, Starling

## Abstract

**Background:**

The present study aimed to evaluate the nutritional proximate composition, some qualitative traits and fatty acid profile of meat from wild thrush, woodcock and starling hunted in Southern Italy in 2017 and 2018.

**Methods:**

Nutritive composition and physical traits of meat and lipid fatty acid profile were evaluated in breast muscle (*Pectoralis major*) of gamebirds.

**Results:**

From findings, the meat pH was significantly (*P* < 0.001) higher in starling when compared to the other two species. Thrush meat was significantly (*P* = 0.002) darker and had higher redness (*P* < 0.001) and yellowness (*P* = 0.004) in comparison to starling and woodcock. Thrush breast muscle showed the highest (*P* < 0.001) level of lipids and lowest (*P* < 0.001) protein content. Meat from thrush showed the best lipid fatty acid profile based on the higher (*P* < 0.001) monounsaturated fatty acids (MUFA) and lower (*P* < 0.001) saturated fatty acids (SFA) concentrations. Starling breast muscle reported the highest (*P* = 0.002) polyunsaturated fatty acids (PUFA) level compared to both thrush and woodcock, whereas no differences were detected on total *n*-3. The ratio *n*-6/*n*-3 was higher (*P* = 0.001) in starling muscle. Thrush breast muscle had the lowest (*P* < 0.001) atherogenic and thrombogenic indices compared to the other gamebirds.

**Conclusions:**

The findings indicated that meat from the three investigated gamebirds species may represent a healthily lipid food source for human consumption in relation to the prevention of cardiovascular diseases.

## Introduction

In the last few years, as a result of the increasing consumer concerns about healthy and safe products, there has been a growing interest in meat from alternative animal species [1, 2]. In the many countries, attention is given to game bird meat as a valuable alternative red- and white-type meat due to the high content of protein, vitamins and trace minerals and the favourable fatty acid profile with high level of polyunsaturated fatty acids (PUFA), and low intramuscular fat content [3]. Moreover, modern consumers have a behaviour essentially related to the safety of products, nutritive value, intrinsic characteristics and taste [4]. In particular, at this regard, the ratio between polyunsaturated and unsaturated fatty acids is believed to be below the optimal values in Western countries, with ruminant meats implicated in this imbalance [5].

The Southern Italy, and in particular in Apulia region, the gastronomic tradition in the autumn-winter period includes several specialties based on wintering avifauna hunting. The thrush *Turdus philomelos*) and woodcock (*Scolopax rusticola*) are among the most representative game bird species hunted in December and January, whereas the hunt of starling (*Sturnus vulgaris*), forbidden in the other Italian regions, has been authorized by derogation in Apulia region in the provinces of Bari and Brindisi during the hunting seasons 2017/2018 in order to restrain the damage caused by these birds on olive crops. Although numerous studies on the meat quality and fatty acid profile have been conducted on poultry species [6], to date the knowledge concerning the chemical composition and nutritive value of game birds meat, such as thrush (*Turdus philomelos*), woodcock (*Scolopax rusticola*) and starling (*Sturnus vulgaris*) is very limited.

Therefore, the aim of this study was to evaluate the nutritional proximate composition, some qualitative traits and fatty acid profile of meat from hunted thrush, woodcock and starling.

## Methods

The study was conducted in Apulia Region (Italy) during the hunting season 2017–2018, collecting three different game birds species commonly hunted: thrush (*Turdus philomelos*), woodcock (*Scolopax rusticola*) and starling (*Sturnus vulgaris*).

A total of sixteen hunted birds for each species were conducted under refrigerated conditions to the Meat Quality Laboratory of Department of Agro-Environmental and Territorial Sciences, University of Bari ‘Aldo Moro’ (Bari, Italy) to conduct chemical and nutritional analyses of meat.

Samples of breast (*Pectoralis major*) muscles were assayed for moisture (945.15), ash (967.05), and crude protein (990.03) by oven, muffle furnace, and Kjeldahl methods, respectively [7]. Total lipids were extracted according to the method of Folch et al. [8]. At 24 h after killing, the breast muscle pH was measured at a depth of 1.0 cm below the surface, using a combined glass-penetrating electrode (Ingold, Mettler Toledo, Greifensee, Switzerland). Color measurements (L* = lightness, a* = redness, b* = yellowness) were assessed on breast (*Pectoralis major* muscle) meat samples using the procedures described by Laudadio et al. [6].

In preparation for fatty acid (FA) composition analysis, samples of breast meat and diet (5 g each) were freeze-dried and then ground. Briefly, methyl heptadecanoate (no. 51633, Fluka, St. Louis, MO) was dissolved into *n*-hexane (1 mg/mL) as an internal standard. Methyl esters of the FA were prepared [9]; samples (300 mg each) and 5 mL of internal standard were incubated (2 h at 80 °C) with methanolic acetyl chloride in a total volume of 9 mL, then the procedures followed those reported by Laudadio et al. [6]. Composition was expressed as percentage of total FA. The atherogenic (AI) and thrombogenic (TI) indexes were calculated according to Ulbricht and Southgate [10] as follows:

$$ \mathrm{AI}=\left(\mathrm{C}12:0+4\times \mathrm{C}14:0+\mathrm{C}16:0\right)/\left[\Sigma \mathrm{MUFA}+\Sigma \left(\mathrm{n}-6\right)+\Sigma \left(\mathrm{n}-3\right)\right]; $$

$$ \mathrm{TI}=\left(\mathrm{C}14:0+\mathrm{C}16:0+\mathrm{C}18:0\right)/\left[0.5\times \Sigma \mathrm{MUFA}+0.5\times \Sigma \left(\mathrm{n}-6\right)+3\times \Sigma \left(\mathrm{n}-3\right)+\Sigma \left(\mathrm{n}-3\right)/\Sigma \left(\mathrm{n}-6\right)\right]. $$

### Statistical analysis

Data were statistically analyzed by a one-way analysis of variance (ANOVA) using a general linear model (GLM) procedure and Duncan’s multiple range test (R Software version 3.4.1). Differences among means were set as significant at *P* ≤ 0.05.

## Results

The proximate nutritional composition and the physical traits of meat from *Pectoralis major* muscle in thrush, woodcock and starling are reported in Table 1. The pH value was significantly (*p <* 0.001) higher in meat from starling (6.38) as compared to woodcock (5.77) and thrush (5.70). The meat from the same species resulted significantly (*P <* 0.001) darker (28.4 vs. 21.4 and 23.1, respectively for starling and woodcock) and had higher redness (15.2 vs 3.7 and 4.7, respectively for starling and woodcock) and yellowness (7.8 vs. 3.6 and 3.9, respectively for woodcock and starling). It was assessed that thrush meat showed the highest amount of fat (4.7 vs. 0.7 and 1.9%; respectively for starling and woodcock; *P <* 0.001), while protein content was the lowest (18.5 vs. 22.2 and 21.7%, respectively for woodcock and starling; *P <* 0.001).

**Table 1:** Nutritional composition and physical characteristics of *Pectoralis major* muscle in thrush, woodcock and starling

Item	Species			SEM	*P*-value
	Thrush (*Turdus philomelos*)	Woodcock (*Scolopax rusticola*)	Starling (*Sturnus vulgaris*)		
pH, at 24 h *post-mortem*	5.70	5.77	6.38	0.03	< 0.001
Moisture, %	73.63	73.88	74.98	0.19	0.065
Protein, %	18.50	22.19	21.72	0.36	< 0.001
Fat, %	4.66	1.85	0.72	0.22	< 0.001
Ash, %	3.20	2.07	1.64	0.07	< 0.001
**Colour measurements**					
L* (lightness)	28.37	23.14	21.38	0.59	0.002
a* (redness)	15.18	4.66	3.74	0.48	< 0.001
b* (yellowness)	7.83	3.63	3.85	0.26	0.004

The lipid fatty acid profile of breast meat in thrush, woodcock and starling are presented in Table 2. Lipid fatty acid profile of meat from thrush showed the best nutritional properties as compared to the other two gamebirds since a significantly (*P <* 0.001) higher concentration of MUFA was recorded (72.4 vs. 47.1 and 49.4%, respectively for starling and woodcock), in turn of a lower SFA content (14.5 vs 36.1 and 32.2%, respectively for woodcock and starling). Meat from starling showed the highest (*P <* 0.001) PUFA level (20.8%) in comparison with thrush (13.5%) and woodcock (14.3%). Further, no difference among gamebirds species regarding the n-3 concentration was detected, whereas meat from starling showed a significantly (*P <* 0.001) greater content of n-6 FA (15.7 vs 8.9 and 9.0, respectively for woodcock and thrush). As a consequence, the *n*-6/*n*-3 ratio was higher (*P <* 0.001) in starling meat (3.5) as compared to woodcock (1.8) and thrush (2.4). In addition, breast muscle from thrush resulted the healthiest because of it showed the lowest (*P <* 0.001) atherogenic (0.08 vs. 0.50 and 0.17, respectively for woodcock and starling) and thrombogenic (0.11 vs. 0.30 and 0.26, respectively woodcock and starling) indices.

**Table 2:** Lipid fatty acid profile of *Pectoralis major* muscle in thrush, woodcock and starling (% on total fatty acids)

Item	Species			SEM	*P*-value
	Thrush (*Turdus philomelos*)	Woodcock (*Scolopax rusticola*)	Starling (*Sturnus vulgaris*)		
C14:0	0.05	0.55	0.28	0.19	< 0.001
C15:0	0.13	0.31	0.17	0.30	0.004
C16:0	8.64	20.18	16.02	0.42	< 0.001
C17:0	0.08	1.66	0.77	0.09	< 0.001
C18:0	5.44	13.13	14.82	0.05	< 0.001
C20:0	0.16	0.24	0.10	0.21	0.005
∑ SFA	14.50	36.06	32.16	0.52	< 0.001
C16:1 *trans*	0.34	0.11	0.33	0.26	< 0.001
C16:1 *cis*	0.44	2.43	1.05	0.06	< 0.001
C17:1	0.04	0.47	0.02	0.09	< 0.001
C18:1 *n*-9 *trans*	0.14	0.33	0.14	0.08	0.0427
C18:1 *n*-9 *cis*	71.24	41.79	42.72	0.13	< 0.001
C24:1 *n*-9	0.18	4.27	2.80	0.12	< 0.001
∑ MUFA	72.38	49.41	47.08	0.40	< 0.001
C18:2 *n*-6 *cis*	7.01	4.37	9.41	0.13	< 0.001
C18:2 *n*-4	0.30	0.26	0.58	0.09	< 0.001
C18:3 *n*-6	0.26	0.47	0.48	0.11	0.009
C18:3 *n*-4	0.18	0.41	0.11	0.38	0.003
C18:3 *n*-3	0.29	2.62	0.64	0.36	< 0.001
C18:4 *n*-3	0.28	0.11	0.71	0.05	< 0.001
C20:3 *n*6	0.43	0.72	0.45	0.06	0.010
C20:4 *n*-6	1.27	2.87	4.66	0.06	0.002
C20:5 *n*-3	0.59	0.98	0.62	0.13	0.287
C22:5 *n*-6	0.06	0.43	0.71	0.12	< 0.001
C22:5 *n*-3	1.46	0.80	0.74	0.05	0.174
C22:6 *n*-3	1.34	0.28	1.73	0.02	0.092
∑ PUFA	13.50	14.30	20.84	0.05	0.002
MUFA + PUFA	85.88	63.72	67.91	0.29	< 0.001
∑ *n*-3	3.97	4.80	4.44	0.12	0.283
∑ *n*-6	9.04	8.84	15.70	0.23	0.001
*n*-6/*n*-3	2.38	1.84	3.53	0.10	0.001
Atherogenic Index	0.08	0.25	0.17	0.11	< 0.001
Thrombogenic Index	0.11	0.29	0.25	0.09	< 0.001

*SFA* Saturated fatty acids, *MUFA* Monounsaturated fatty acids, *PUFA* Polyunsaturated fatty acids; n-6/n-3, PUFA n-6/PUFA n-3 ratio

## Discussion

In the present study, meat nutritional and physical characteristics as well as the lipid fatty acid (FA) profile differed significantly among the investigated gamebirds. We could not locate any literature concerning the characteristics of meat lipid fatty acid composition of thrush, woodcock and starling; therefore, this subject should be considered as a new investigation. It is well know that the FA composition of muscles reflected the FA composition of diets [5], and this aspect is particularly evident in avian species [11–13], including gamebirds. The results from the present study may be due to the different eating habits of thrush which prefers berries and fruits instead of insects that, on the other hand, are the main feeding source for woodcock and starling.

Dietary lipids are well known to have a key-role on cell membrane, tissues and meat lipid profile as well as on plasma lipoproteins depended on beneficial fatty acid level. Because of the health positive effects linked with bioactive compounds, further studies are needed to improve different animal-origin foods [14]. In the present study, this healthy property could be related to the high PUFA content in meat from gamebirds.

Although the ratio SFA to MUFA represents an significant factor from a human diet viewpoint, particular SFA and PUFA have diverse metabolic effect [15]. The FA can either support or avoid coronary thrombosis and atherosclerosis, due to the direct influence on serum total cholesterol and LDL-cholesterol levels. Therefore, the AI was defined [10]. In the present research, the AI of the gamebirds meat lipids varied significantly among species.

The meats from gamebirds investigated were characterised by a low AI value, which were related to the low ratio SFA to MUFA. Low levels of AI is recommended for a healthy diet [15]. At this regards, the fatty acids C14:0 and C16:0 are well known to have an atherogenic effect, conversely C18:0 results neutral in relation to the atherogenicity, however it is has a thrombogenic effect [16]. An additional positive characteristic derived by meat lipid profile in this study was the low levels for both AI and TI, when compared to other animal derived products [15, 17].

## Conclusions

The findings of this study indicated that meat from game birds can represent a valuable food providing a valuable lipid fatty acid profile and healthy balanced diets that can prevent human diseases related to the cardiovascular system. However, further research needs to be conducted to deeply define di nutritional and healthy potential and consumers perception of gamebirds meat as alternative food resources.

## Data Availability

All data generated or analyzed during this study are included in this article.

## Abbreviations

AI: Atherogenic index
LDL: Low-density lipoproteins;
MUFA: Monounsaturated fatty acids
n-6/n-3: PUFA n-6 to PUFA n-3 ratio
PUFA: Polyunsaturated fatty acids
SEM: Standard error of the means
SFA: Saturated fatty acids
TI: Thrombogenic index
